# Desafíos de la gobernanza del sistema de salud y lecciones del covid-19 para Colombia

**DOI:** 10.15446/rsap.V25n5.111796

**Published:** 2023-09-01

**Authors:** Natalia Arias-Monsalve, Jairo H. Restrepo-Zea

**Affiliations:** 1 NA: Politóloga. M. Sc. Políticas Públicas. Consultora e Investigadora. Medellín, Colombia. natalia.ariasml@udea.edu.co Políticas Públicas Consultora e Investigadora Medellín Colombia natalia.ariasml@udea.edu.co; 2 JR: Econ. M. Sc. Gobierno y Asuntos Públicos. Profesor titular y coordinador del Grupo de Economía de la Salud (GES). Facultad de Ciencias Económicas, Universidad de Antioquia. Medellín, Colombia. jairo.restrepo@udea.edu.co Universidad de Antioquia Grupo de Economía de la Salud (GES) Facultad de Ciencias Económicas Universidad de Antioquia Medellín Colombia jairo.restrepo@udea.edu.co

**Keywords:** Gobernanza, sistema de salud, marco de evaluación, covid-19, Colombia *(fuente: DeCS, BIREME)*, Governance, health systems, evaluation framework, covid-19, Colombia *(source: MeSH, NLM)*

## Abstract

**Objetivo:**

Determinar la calidad de la gobernanza del sistema de salud de Colombia y analizar las lecciones que trajo la pandemia del covid-19, teniendo en cuenta la percepción de líderes que hacen parte del sistema.

**Material y Métodos:**

Estudio descriptivo y exploratorio basado en datos de encuesta propia, cuya muestra fue no probabilística por conveniencia. Participaron 107 líderes del sistema de salud. Los datos se analizaron considerando un marco de evaluación de desempeño de los sistemas de salud.

**Resultados:**

Los participantes en el estudio calificaron la gobernanza del sistema de salud en un promedio de 2,6/5,0, lo que corresponde a una calidad baja de la gobernanza. No obstante la baja calificación, los líderes percibieron mejoras durante la pandemia en el desempeño de propósitos como la visión estratégica y la supervisión del sistema, y sobre las capacidades consideraron mejoras en el manejo de riesgos y la gestión de la información.

**Discusión:**

Este estudio brinda una aproximación sobre la gobernanza del sistema, considerando las subfunciones propuestas en el marco de evaluación, lo que constituye una oportunidad para conocer las fallas y los aspectos en que se debe mejorar, aún más, luego de pasar por las presiones del covid-19, por lo que el sistema debería prepararse para un próximo choque externo. La investigación ofrece un panorama para nuevos estudios que puedan usar el mismo marco y proponer indicadores de gobernanza para evaluarla y realizar seguimiento.

## Abstract (en)

**Objective:**

To determine the quality of governance of the Colombian health system and to analyze the lessons brought by the covid-19 pandemic, considering the perception of leaders who are part of the system.

**Material and Methods:**

Descriptive and exploratory study based on survey data, whose sample was non-probabilistic for convenience. 107 health system leaders participated. The data were analyzed considering a health systems performance evaluation framework.

**Results:**

Survey participants rated health system governance an average of 2.6/5.0, corresponding to low quality of governance. Despite the poor rating, leaders perceived improvements during the pandemic in the performance of purposes such as strategic vision and system supervision, and regarding they considered improvements in capabilities of risk management and information management.

**Discussion:**

This study provides an approach to the governance of the system, considering the subfunctions proposed in the evaluation framework, which constitutes an opportunity to know the failures and aspects that must be improved, even more, after going through the pressures of the covid-19. The system must learn from these aspects to be prepared for the next external shock. The research offers an outlook for new studies that can use the same framework and propose governance indicators to evaluate and monitor it.

Para la Oficina del Alto Comisionado de las Naciones Unidas para los Derechos Humanos (ACNUDH) 1, la buena gobernanza en un Estado radica en el grado en que los procesos y los resultados políticos e institucionales cumplen con los derechos humanos (civiles, culturales, políticos y sociales). En el caso de la salud, la buena gobernanza se vincula con el cumplimiento de la cobertura universal, servicios de salud de calidad y brindados con oportunidad. Sin embargo, estudiar la gobernanza y conocer su calidad y desempeño, es esencial para los sistemas de salud, pues da forma a la capacidad de estos para responder a problemas, desafíos cotidianos y nuevas políticas 2. Además, la calidad de la gobernanza afecta la capacidad del sistema de salud para ser sostenible, universal y de alta calidad 2.

Para Lewis 3, contar con una buena gobernanza radica en que los servicios de salud se deriven de la eficiencia de los recursos financieros, humanos y de suministro, y de la prestación de los servicios distribuidos espacialmente en todo el país de manera oportuna. Por otra parte, la buena gobernanza crea un contexto para proteger los derechos humanos. Según la ACNUDH, dicho contexto "comprende los marcos jurídicos y las instituciones adecuadas, así como los procesos políticos, administrativos y de gestión necesarios para responder a los derechos y las necesidades de la población" 1. Por estos motivos, cada vez toma más relevancia la gobernanza en los sistemas de salud.

Aunque no se cuenta con una definición única de gobernanza en los sistemas en salud, la evolución del concepto muestra elementos comunes y descripciones de los atributos que son inherentes a ella. Existe algún consenso en cuanto a que la gobernanza abarca un "conjunto de procesos (costumbres, políticas o leyes) que se aplican formal o informalmente para distribuir la responsabilidad o la rendición de cuentas entre los actores de un determinado sistema de salud" 4, y comprende: la rendición de cuentas, la representación, la administración, la propiedad, el poder, la autoridad y el estado de derecho.

Estos elementos comunes también se ven reflejados en los indicadores propuestos para evaluar la gobernanza. Algunos autores han desarrollado marcos de análisis y evaluación, como aquellos destinados a analizar la calidad de la gobernanza en salud. Es el caso del Marco Analítico de Gobernanza (MAG), de Marc Hufty 5, el Marco TAPIC (transparencia, rendición de cuentas, participación, integridad y capacidad, por sus siglas en inglés), de Greer *et al.*^2^, y el Marco de Evaluación de Desempeño de los Sistemas de Salud, de Papanicolas *et al.*^6^. En varios estudios se encuentra el uso de este tipo de marcos para analizar la gobernanza en países específicos, aunque no se tienen muchos estudios para la región de América Latina.

Conocer la calidad de la gobernanza de un sistema de salud es indispensable para alcanzar sus metas y propósitos, además de ser un activo fundamental al momento de enfrentar choques externos que lo afectan, como las pandemias y los problemas económicos, políticos y sociales. De manera general, la Organización Panamericana de la Salud (OPS) 7 ha mencionado que la pandemia del covid-19 y su impacto en los sistemas de salud ha vuelto más visible o ha exacerbado las debilidades estructurales de los sistemas de salud. En tal sentido, recomienda "fortalecer el liderazgo, la rectoría y la gobernanza con un énfasis renovado en las funciones esenciales de salud pública" 7.

Colombia no escapó a la visibilidad de las debilidades del sistema de salud, las cuales habían sido documentadas desde antes de la pandemia del covid-19 8. El sistema colombiano se caracteriza por ser financiado públicamente, basado en el aseguramiento y con la participación de diferentes agentes en la gestión del seguro de salud y en la prestación de los servicios 9, lo que implica una actuación de múltiples actores con diferentes intereses, que no siempre llegan a acuerdos en la toma de decisiones. En este sentido, cobra importancia hablar de la calidad de la gobernanza en el sistema de salud.

Sobre las aproximaciones de gobernanza en el país, se destacan los estudios de Roth y Molina 10 y de Restrepo y Zapata 11, en los cuales se analizan las debilidades relacionadas con la gobernanza del sistema. El primero en mención concluyó que la toma de decisiones en salud pública es débil, como consecuencia de la frágil capacidad rectora de la autoridad sanitaria, lo cual se atribuye al poco desarrollo y la baja capacidad institucional, la fragmentación de las responsabilidades y las competencias, la interferencia de intereses particulares y clientelares en la elaboración y la implementación de la normatividad, así como en los procesos clave de la política y la gestión en salud. Por su parte, Restrepo y Zapata argumentan que la gobernanza en el sistema es débil, dada la calificación que realizaron diferentes actores (2,3/5,0), y señalaron principalmente problemas de corrupción, falta de rendición de cuentas, escasa participación y falta de coordinación intersectorial.

Aunque en Colombia han existido organismos para desarrollar algunas funciones relacionadas con la gobernanza del sistema, como la regulación, la administración financiera y la concertación, estos organismos han resultado inestables en el marco de la evolución del sistema 9 y han carecido de modelos de gobernanza que impliquen la participación de todos los actores o sus representantes y la toma de decisiones colectivas. En tal contexto, este estudio busca analizar la calidad de la gobernanza en el sistema de salud colombiano, considerando la percepción de sus líderes, en el marco de evaluación de desempeño de los sistemas de salud de Papanicolas *et al.*^6^, ya que se trata de un marco reciente, el cual no se ha usado para el caso de Colombia.

El marco de evaluación de desempeño identifica y describe las funciones del sistema de salud (gobernanza, financiamiento, prestación de servicios y generación de recursos), proporciona un marco para evaluar cada función y relaciona el desempeño con los objetivos y las metas del sistema. En lo que respecta a la gobernanza, esta se concibe como la función primordial, transversal y habilitadora, que debe operar en las otras funciones. Así, se habla de gobernanza del financiamiento, gober-nanza de la generación de recursos y gobernanza de la prestación de servicios, entendiendo que existe una gobernanza general del sistema.

Finalmente, este estudio busca conocer si se presentaron cambios en la gobernanza debido a la pandemia del covid-19, y en tal caso, si se encuentran ejemplos y referentes para que ella mejore. Conocer los desafíos y las lecciones de gobernanza aporta información que sirve para mejorar su desempeño, los atributos que la componen, como la transparencia, la rendición de cuentas y la participación, y los procesos de toma de decisiones que impacten favorablemente al sistema de salud y a la población del país.

## MATERIAL Y MÉTODOS

Se llevó a cabo un estudio descriptivo y exploratorio basado en información de fuentes primarias, a partir de una encuesta con muestra no probabilística por conveniencia, sobre la opinión de la gobernanza. Se contó con la participación de 107 líderes del sistema de salud, entre 302 que fueron convocados, según el siguiente perfil: persona que por su trayectoria o posición ejerce responsabilidades o liderazgo frente al desempeño del sistema y cuyas opiniones reflejan el sentir de estamentos, gremios o sectores, y contribuyen a formar la opinión de sus representados y de la ciudadanía.

Los líderes participantes están vinculados a: sector público, sector privado, academia, gremios de profesionales, industria, organizaciones de pacientes o usuarios, asociaciones científicas, instituciones prestadoras de salud (IPS), organizaciones comunitarias, entidades prestadoras de salud (EPS) y medios de comunicación. Los líderes contactados hacían parte de la base de datos de contactos y mapeo de actores del Grupo Economía de la Salud (GES) de la Facultad de Ciencias Económicas de la Universidad de Antioquia.

La encuesta consistió en 22 preguntas que indagaron por la gobernanza en el sistema de salud y la caracterización sociodemográfica de los encuestados (Cuadro 1).

**Cuadro 1:** Secciones de la encuesta de gobernanza a líderes del sistema de salud

Secciones	Relación con las subfunciones de gobernanza	Preguntas
Valoración de la gobernanza	Visión y política	¿Qué tan buena es la gobernanza en el sistema de salud? Enuncia las debilidades y fortalezas de la gobernanza del sistema. Indique si las instancias o escenarios influyen en la toma de decisiones del sistema (10 instancias o escenarios).
Lecciones de la gobernanza durante la pandemia del covid-19	Visión y política, voz de las partes interesadas, información e inteligencia, legislación y regulación	Califique el desempeño de las siguientes funciones (10 funciones) del sistema de salud durante la pandemia del covid-19 (mejoró, empeoró o siguió igual). Califique el comportamiento de los actores en materia gobernanza durante la pandemia (mejoró, empeoró o siguió igual). ¿Como califica las capacidades (9 capacidades) que ha desarrollado el país en los últimos dos años para enfrentar una situación como la pandemia? (mejoró, empeoró, siguió igual).
Desafíos y oportunidades	Visión y política, voz de las partes interesadas, información e inteligencia, y legislación y regulación	¿Cree que se podría mejorar la gobernanza en el sistema de salud colombiano? ¿Cómo se podría mejorar la gobernanza? De la siguiente lista de problemáticas del sistema de salud, seleccione tres que deben ser intervenidas con urgencia.
Caracterización sociodemográfica	Voz de las partes interesadas	Edad, sexo, ciudad de residencia, grado de escolaridad, profesión, cargo u ocupación, sector que representa.

El diseño de las preguntas se basó en el estudio realizado por Restrepo y Zapata 11, y se tuvieron en cuenta algunos de los criterios de las áreas de evaluación de las subfunciones de gobernanza propuestas en el marco de evaluación adoptado 6. Vale aclarar que las subfunciones inherentes a la gobernanza son: visión y política, voz de las partes interesadas, información e inteligencia, legislación y regulación.

Los datos obtenidos se analizaron para establecer la calidad de la gobernanza en general, según los actores, y para establecer los datos que guardaban relación con las subfunciones de gobernanza. Para iniciar la encuesta se brindó una definición de gobernanza 11, a fin de evitar que los participantes se confundieran con otros conceptos como el de gobernabilidad, institucionalidad, legitimidad.

La encuesta se llevó a cabo utilizando el *software* de Typeform, a través de un enlace electrónico que conducía a un cuestionario digital, desde computador, teléfono inteligente o tableta. Los datos obtenidos se organizaron en el programa Excel, y las preguntas abiertas se organizaron según categorías o atributos de la gobernanza. El formato de la encuesta se basó en el autodiligenciamiento de manera anónima; al final se solicitó el correo electrónico en caso de querer recibir los resultados.

Con respecto a la gobernanza y las lecciones del covid-19, se contemplaron preguntas sobre las capacidades y el desempeño de algunos propósitos del sistema durante la pandemia. Asimismo, se indagó por los modos de gobernanza que surgieron para dar respuesta a la pandemia, como las estrategias o reglas informales entre los agentes (Cuadro 2).

**Cuadro 2:** Subfunciones de la gobernanza

Subfunción	Características	
Visión y política	Capacidad y recursos necesarios para proporcionar una visión estratégica, articulada con las políticas y leyes, por las cuales los gobiernos rinden cuentas/existencia de la colaboración intersectorial.	Rendición de cuentas y agencia Instituciones aptas para los propósitos del sistema (instituciones funcionales y diseño institucional) Transparencia (disponibilidad pública de la información utilizable)
Voz de las partes interesadas	Participación de las partes interesadas, como la academia, proveedores, sociedad civil, comunidades vulnerables, para contribuir en las decisiones de políticas de salud/equilibrio en relaciones de poder/mecanismos institucionalizados/creación de consenso/plataformas de diálogo funcional.	
Información e inteligencia	Recopilación, análisis y uso de datos, información e inteligencia de y para el sistema de salud/accesibilidad de la información para las partes interesadas/gerenciamiento y voluntad política.	
Legislación y regulación	Capacidad institucional para la regulación y el cumplimiento en salud/cumplimiento de la legislación y reglamentación por parte de los actores/incentivos.	

Fuente: Datos de Papanicolas et al. 6.

## RESULTADOS

La encuesta permitió ratificar el carácter de líderes de los participantes, pues el 59,8% informó que tenía un nivel de liderazgo de 4, en una escala de 1 a 5, siendo 5 el mayor nivel de liderazgo; el 29% definió su liderazgo en 5, y el 10,3% en 3. En lo concerniente al sector del que hacen parte estos líderes, el 58,9% está vinculado al sector público, el 11,2 % a la academia, el 6,5% a los gremios profesionales y a EPS. Sobre el grado de escolaridad, el 82% cuenta con estudios de posgrado, mientras que un 15% tiene pregrado, y un 3% cuenta nivel técnico o tecnológico. Principalmente, el cargo u ocupación señalado fue: profesional en una entidad pública (32%), directivo de segunda línea de una entidad pública (15%), gerente, director o presidente de una entidad pública (14%).

La definición suministrada en la encuesta, para valorar la calidad de la gobernanza, fue tomada del estudio de Restrepo y Zapata: "Una forma de gobernar el sector de la salud que se caracteriza por la participación y la coordinación de actores, la toma de decisiones y la implementación de políticas públicas, de manera negociada y bajo una rectoría que busca el cumplimiento de un objetivo común: garantizar el derecho a la salud de la población mediante la prestación de los servicios de salud con eficiencia, suficiencia y calidad" 11.

**Figura 1:**
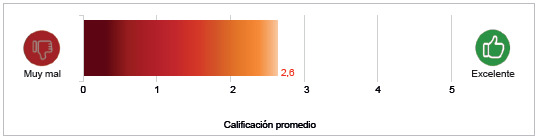
¿Qué tan buenaeslagobernanzadelsistemadesaludcolombiano?

Con esta definición como marco, en una escala de 1 a 5, los participantes asignaron una calificación según qué tan buena es la gobernanza en el sistema de salud. De esta manera, se obtuvo la calificación promedio de la gobernanza de 2,6/5,0 (Figura 1). Esta calificación indica una calidad baja de la gobernanza en el sistema.

Para dar cuenta de las razones de la baja calidad de la gobernanza, los participantes respondieron de manera abierta por las fortalezas y debilidades. Con respecto a las fortalezas, mencionaron principalmente: la normatividad, la cobertura en salud y la experiencia del sistema. Sobre las debilidades, señalaron principalmente tres: la corrupción, la escasa participación ciudadana en la toma de decisiones del sistema y la inadecuada gestión de los recursos financieros. Las debilidades y fallas aquí señaladas sugieren que las subfunciones de la gobernanza no operan de manera apropiada (Cuadro 3).

**Cuadro 3:** Fallas en las subfunciones de la gobernanza

Subfunciones	Debilidades relacionadas con las subfunciones de gobernanza
Voz de las partes interesadas	Corrupción Baja participación Toma de decisiones sin concertación entre diferentes actores Desconfianza
Visión y política	Baja rectoría y liderazgos Falta de visión común o compartida Baja coordinación Baja articulación
Información e inteligencia	Escasa rendición de cuentas Falta de transparencia Baja Información
Legislación y regulación	Exceso de normatividad Falta de definición de roles y responsabilidades

Los participantes comentaron que la gobernanza no funciona bien y es de mala calidad porque existen intereses particulares de los actores que se traducen en prácticas corruptas, hay una débil vigilancia y sanción en el sistema, y falta mayor información y transparencia en las decisiones que lo afectan. A esto se suma la falta de espacios para la participación real, es decir, espacios que tengan en cuenta con igual peso en el voto, las decisiones y las opiniones de todos los actores con relación a un tema en particular. Se destaca también la falta de articulación y coordinación entre diferentes niveles del sistema. Nótese que en todas las subfunciones de gobernanza se destacaron debilidades, y debe tenerse en cuenta que no puede hablarse de una subfunción más importante que otra, ya que se encuentran interrelacionadas.

**Figura 2:**
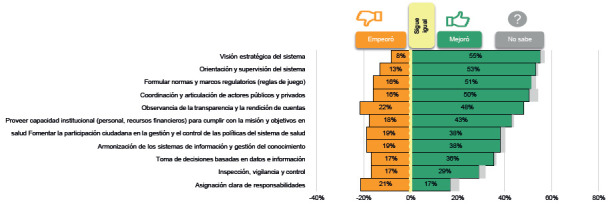
Desempeño de los propósitos de las subfunciones de gobernanza frente al covid-19

De otro lado, para comprender las lecciones de gobernanza que pudo traer consigo el covid-19, se preguntó a los participantes por el desempeño de algunos propósitos relacionados con las subfunciones de gobernanza, considerando si cada propósito empeoró, mejoró o siguió igual a causa de la pandemia. Se destaca el optimismo sobre mejoras en la visión estratégica y la orientación y supervisión del sistema, las reglas del juego y la coordinación y articulación entre actores públicos y privados, entre otros propósitos (Figura 2). Sin embargo, también se reconocieron aspectos que empeoraron durante la crisis, como la transparencia y la rendición de cuentas y la provisión de capacidad institucional. Estos últimos están relacionados con la subfunción de información e inteligencia y la legislación y regulación, y en general los propósitos que empeoraron guardan relación con las debilidades mencionadas antes, como la corrupción, las fallas en la información y la desconfianza entre actores.

**Figura 3:**
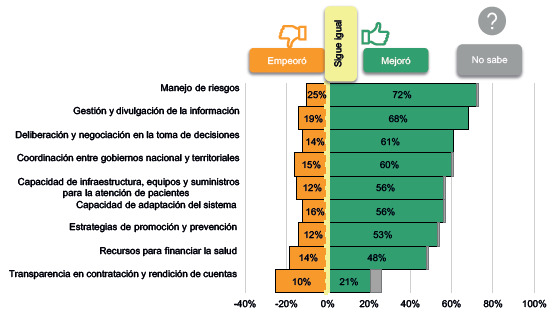
Capacidades que se desarrollaron en el país para enfrentar la pandemia del covid-19

De otro lado, al indagar por las mejoras en las capacidades se obtuvo que el "manejo de riesgos", la "capacidad de infraestructura, equipos y suministros" y la "gestión y divulgación de la información" presentaron mejoras durante la pandemia. Los aspectos que empeoraron fueron la "transparencia en la contratación y rendición de cuentas", seguido por las "estrategias de promoción y prevención" (Figura 3).

Ahora bien, en contraste con las Figuras 2 y 3, se dieron mejoras en la "capacidad de infraestructura, equipos y suministros" (por ejemplo, aumento de cama de cuidados intensivos) y en proveer la "capacidad institucional (personal, recursos financieros) para cumplir con la misión y objetivos en salud". Sin embargo, un 22 % señaló que esta última capacidad empeoró debido a la falta de personal o recurso humano para atender la pandemia y el resto de los servicios que no tenían relación con enfermedad del covid-19. Otro contraste interesante es que en ambas figuras se muestra que empeoró el propósito relacionado con la transparencia y la rendición de cuentas.

Asimismo, se indagó por las estrategias de gobernanza para dar respuesta a la enfermedad del covid-19. Esencialmente, se indicó que la toma de decisiones fue más rápida y se dio mayor rapidez en la transferencia de recursos, se compartieron conocimientos, espacios para la articulación de la red privada y la red pública para la transparencia. También se adujo que la información fue más abierta, y en algunas estrategias de contención de la pandemia se dieron procesos de participación que incluyeron a la comunidad.

Es de señalar que el 97 % de los encuestados consideraron que la gobernanza del sistema puede mejorar, y para ello adujeron que es necesario contar con: coordinación entre actores (con resultados concretos), una participación efectiva en la toma de decisiones, una vigilancia y control fortalecida, mayor transparencia en el manejo de los recursos financieros, reales sanciones para los actores corruptos, mejorar en la construcción de la confianza entre actores, y contar con un sistema de información eficiente y articulado.

## DISCUSIÓN

Los resultados de este estudio brindan una radiografía sobre cómo es la calidad de la gobernanza en el sistema de salud, por lo que fue posible establecer un nivel bajo (2,6/5,0), y esto implicó que los actores reconocieran fallas en aspectos inherentes a la función de la gobernanza como la participación, la transparencia (señalando la corrupción), la rendición de cuentas, la información, la gestión de recursos y la coordinación.

Lo anteriormente señalado permite comprender que existen debilidades en las subfunciones de la gobernanza: "visión y política", "voz de las partes interesadas", "información e inteligencia", y "legislación y regulación", lo que a la vez indica que las áreas de evaluación propuestas en el marco, es decir, la transparencia, la rendición de cuentas y las instituciones aptas para los propósitos del sistema no se desempeñan bien. Este panorama es de importante atención, pues como explican Greer *et al.*^2^, "la calidad de la gobernanza afecta la capacidad del sistema de salud para ser sostenible, universal y de alta calidad y, en general, puede afectar la capacidad de toda una sociedad para buscar bienes sociales".

Es de notar que tanto la participación como los espacios para la toma de decisiones resultaron ser cuestionados, porque las personas que logran participar influyen solo de manera moderada en las decisiones, lo que puede explicar una falta de horizontalidad o equilibrio en el poder de los actores, e inclusión y concertación para la toma de decisiones. Esto deriva en una necesidad del sistema de contar con un espacio o mecanismo de participación que involucre a todos los actores o sus representantes en la toma de decisiones.

En palabras de Figueras 12, ni la centralización ni la descentralización son mejores, simplemente lo mejor ha sido la transparencia y la coordinación. Importa menos dónde se ha tomado la decisión, sino que haya sido alineada y concertada.

Las lecciones aprendidas a partir de las estrategias para enfrentar el covid-19 deben servir de base para mejorar la gobernanza. Las lecciones han sido ganancias que no deberían perderse, como el manejo de riesgos, la capacidad de infraestructura, equipos y suministros para atención de pacientes y la articulación e información entre actores. Un ejemplo de ello fue la regulación de camas de Unidad Cuidados Intensivos UCI en Bogotá. Un estudio realizado por Álvarez *et al.*^13^ da cuenta sobre la gestión de las camas UCI durante la pandemia por el Centro Regulador de Urgencias y Emergencias (CRUE). Este centro organizó las respuestas frente a la alta demanda de camas y creó una estrategia de coordinación entre diferentes agentes del sistema (una de varias estrategias), como las IPS, que debían mantener comunicación directa con el CRUE además de sumarse las ambulancias de las Empresas Administradoras de Planes de Beneficios (EAPB), todo ello soportado por los actores en el Sistema Integral de Referencia y Contrarreferencia. La gestión centralizada de las camas favoreció la atención oportuna de los pacientes, lo que redujo las barreras administrativas de acceso a servicios especializados 13.

Aunque en algunos casos mejoró la gobernanza del sistema de salud, persiste la necesidad de visibilizar y adoptar estas estrategias en el día a día del sistema. El centro de pensamiento Así Vamos en Salud recomienda, en relación con la gobernanza y la pandemia, la estrategia de "Desarrollar un espacio para compartir experiencias aprendidas durante la pandemia por COVID-19" 14.

De otro lado, los aspectos que empeoraron durante la crisis, como la "Observancia de la transparencia y la rendición de cuentas" (21%) y "Proveer capacidad institucional (personal, recursos financieros) para cumplir con la misión y objetivos en salud", señalada por el 22 % de los participantes, brindan información sobre qué aspectos priorizar y focalizar para que la gobernanza mejore. La gobernanza como función habilitadora y transversal redunda en las mejoras de las otras funciones fundamentales del sistema. Según el *European Observatory on Health Systems and Policies*^15^, la gobernanza es un facilitador para liderar un sistema de salud en tiempos de emergencia, evitando que se conviertan en una crisis.

Los anteriores hallazgos son coherentes con lo evidenciado por Roth y Molina 10 sobre cómo la toma de decisiones en la salud pública en el sistema de salud de Colombia es débil, debido a que la capacidad rectora de la autoridad sanitaria sigue siendo frágil.

Asimismo, cabe recalcar que existen coincidencias de los resultados aquí expuestos con lo que encontraron Restrepo y Zapata 11. Se destaca que los actores calificaron la gobernanza general del sistema como baja, con una calificación de 2,3/5,0, mientras que en este estudio el resultado fue 2,6/5,0. De manera similar, se reconocieron las fortalezas del sistema, como la cobertura, el plan de beneficios, los recursos financieros y el desarrollo normativo y regulatorio. También se mencionó la importancia de fortalecer los procesos de rendición de cuentas, la transparencia en la toma de decisiones, y contar con medidas para la evaluación del funcionamiento de las instancias de participación, lo cual coincide con este estudio y con las opiniones de los actores.

Conocer las fortalezas y las debilidades de la gobernanza del sistema puede servir para marcar el camino hacia la acción y resistir mejor los impactos negativos de las crisis. Tener en cuenta la información dada por los actores, como la que aquí se presenta, es fundamental para una política pública que busque reformar el sistema de salud. La información sobre los problemas o las fallas en la gobernanza indica dónde se deben focalizar las acciones para mejorar la gobernanza, lo que ayudaría a consolidar un modelo e instancia de gobernanza que cuente con voz y voto de los actores para la toma de decisiones de manera informada y consensuada, lo cual a su vez, como mencionan Restrepo y Zapata, "aporta a las metas de lucha contra la corrupción en cuanto desde estas se puede hacer vigilancia y control social, superar las fallas de coordinación y a mejorar la relación Estado-sociedad por medio de la participación ciudadana" 11.

En todo lo anterior, es importante tener en cuenta los planteamientos de Sagan *et al.*^16^, en cuanto un país que tiene sistemas efectivos de gobernanza tiene más probabilidad de responder de manera efectiva en una crisis y, por lo tanto, limitar el daño a la salud y la economía ♦
